# Design of the DEMO Fusion Reactor Following ITER

**DOI:** 10.6028/jres.114.016

**Published:** 2009-08-01

**Authors:** Paul R. Garabedian, Geoffrey B. McFadden

**Affiliations:** Courant Institute, New York University, 251 Mercer St., New York, NY 10012; Mathematical and Computational Sciences Division, National Insitute of Standards and Technology, Gaithersburg, MD 20899

**Keywords:** computational science, magnetic fusion, plasma physics

## Abstract

Runs of the NSTAB nonlinear stability code show there are many three-dimensional (3D) solutions of the advanced tokamak problem subject to axially symmetric boundary conditions. These numerical simulations based on mathematical equations in conservation form predict that the ITER international tokamak project will encounter persistent disruptions and edge localized mode (ELMS) crashes. Test particle runs of the TRAN transport code suggest that for quasineutrality to prevail in tokamaks a certain minimum level of 3D asymmetry of the magnetic spectrum is required which is comparable to that found in quasiaxially symmetric (QAS) stellarators. The computational theory suggests that a QAS stellarator with two field periods and proportions like those of ITER is a good candidate for a fusion reactor. For a demonstration reactor (DEMO) we seek an experiment that combines the best features of ITER, with a system of QAS coils providing external rotational transform, which is a measure of the poloidal field. We have discovered a configuration with unusually good quasisymmetry that is ideal for this task.

## 1. Introduction

A hot plasma of hydrogen isotopes can be confined in a strong magnetic field with toroidal geometry so the ions fuse to form helium and release energetic neutrons. Models of such magnetic fusion devices developed in computational science have been implemented as codes that comprise an effective numerical simulation of the most essential features of modern tokamak and stellarator experiments. This has led to the discovery of advanced concepts that make fusion reactors a realistic prospect for a commercial source of energy. Here we apply the NSTAB and TRAN computer codes to issues of equilibrium, stability and transport that provide an example of the theory [8, 16].

When the mesh size of the computational grid for fusion calculations is comparable to the width of the islands, an exceptionally accurate radial difference scheme in conservation form enables one to capture them successfully despite a nested surface hypothesis imposed by the mathematics [2]. Asymmetries in bifurcated 3D solutions of the axially symmetric tokamak problem are examined to ascertain their relevance to the observation of neoclassical tearing modes (NTMs) and ELMs in experiments. Compact stellarator equilib-ria with quasiaxial symmetry are calculated to see whether physical advantages of these 3D configurations might support the feasibility of magnetic fusion if troubles with safety and stability occur in the ITER project [14].

## 2. Conservation Form

Let us consider the magnetohydrodynamic (MHD) model of plasma physics. We have ∇ ⋅ **B** = 0, where **B** is the magnetic field. The requirement of force balance put on the plasma follows from the simplified form

$$\int \int \int \left(\frac{1}{2}B^{2}-p\right)dV=\min\text{imum}$$

of the MHD variational principle, where *p* is the fluid pressure and the gas constant is zero. In a stable system the energy throughout the volume of plasma must be a minimum [4,10]. A mathematical consequence of this principle asserts that the Lorentz force must balance the pressure, so that

∇⋅T=0,∇⋅B=0,

where **T** is the Maxwell stress tensor

$$T=\mathrm{BB}-\left(B^{2}/2+p\right)I.$$

The divergence theorem can be applied to the MHD equations over any test volume of the plasma because we have put them in a conservation form with each term expressed as an exact partial derivative. It follows that the force and the normal component of the magnetic field do not have jumps across any discontinuity of the plasma such as might occur at a free boundary or in a weak solution satisfying integrated conservation laws rather than differential equations. If **N** is the normal, the result can be stated more precisely in the form

$$B\cdot N=0,\;\left[B^{2}/2+p\right]=0,$$

where the square brackets indicate that a jump is to be calculated.

**Fig. 1:**
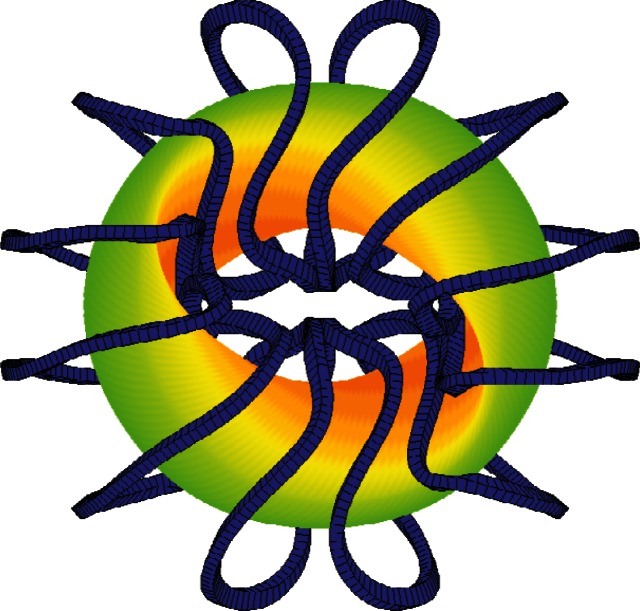
Magnetic fusion of hydrogen forms helium and emits energetic neutrons from a torus of plasma shaped to optimize confinement. Modular coils generate a field keeping the ions from hitting walls.

In selecting a desirable stellarator configuration a first step is to find a representation in cylindrical and toroidal coordinates for the free boundary

$$r+iz=e^{iu}\sum \Delta_{mn}\;e^{-imu+inv}$$

that optimizes physical properties of the plasma inside. After the shape of the plasma has been found one must determine positions of coils on an outer control surface defined by an expression like the one for the free boundary. These coils should be smooth and should be constructed by a filtered form of the Biot-Savart law to generate an external magnetic field, consistent with the one defined by current inside the plasma, that has robust flux surfaces devoid of extraneous harmonics [1]. Attractive coils producing the desired field have been found even when their distance from the boundary is large in units of the plasma radius (cf. Fig. 1).

## 3. Force Balance

The NSTAB code captures islands correctly despite a nested surface hypothesis made in the numerical algorithm employed. Partial differential equations are solved in a conservation form described above that calculates force balance correctly across islands that are treated as discontinuities. The reliability of the code has been established by using it to study zero pressure stellarators where islands are known to exist in equilibria found by solving the Laplace equation. In calculations of this kind the rotational transform *ι* can change sign so that a sizeable island appears where it vanishes.

In the NSTAB code we implement a continuum model of plasma physics that is defined by differential equations for the magnetic field **B**, the current density **J**, and the fluid pressure *p*. The Maxwell stress tensor **T** has been employed so that analogous finite difference equations have the advantage that when they are summed over a test volume they telescope into an approximate statement of force balance across the boundary that is globally correct. Numerical calculations of weak solutions that capture discontinuities this way at resonant surfaces in plasma equilibria serve to simulate significant aspects of the physics that are hard to analyze by other methods.

The theory can be justified by considering the simplified example of the Burgers equation [3]

$${2\psi }_{x}\psi_{xx}={\left(\psi_{x}^{2}\right)}_{x}=\eta \psi_{xxx},$$

subject to three boundary conditions Ψ(− 1) = Ψ(1) = 0, Ψ*_x_*(− 1) = 1. This models a reversed field pinch (RFP) depending on only one coordinate *x*, where Ψ is the flux, Ψ*_x_* is the principal component of the magnetic field, Ψ*_xx_* is the current, and *η* is an artificial resistivity. The conservative difference scheme

$$\begin{array}{l} {\left(\psi_{n+1}-\psi_{n}\right)}^{2}-{\left(\psi_{n}-\psi_{n-1}\right)}^{2}=\\ \eta \left(\psi_{n+2}-3\psi_{n+1}+3\psi_{n}-\psi_{n-1}\right) \end{array}$$

for the Burgers equation computes jumps across discontinuities correctly and therefore imposes force balance across a sharp boundary which occurs at *x* = 0 in the limiting case where *η* = 0 so that the solution reduces to Ψ = 1 − |*x*|.

Let us consider a finite difference scheme for the RFP problem that is not in conservation form and is defined by the equation

$$\begin{array}{l} {\left(\psi_{n+1}-\psi_{n}\right)}^{2}-{\left(\psi_{n}-\psi_{n-1}\right)}^{2}+\varepsilon {\left(\psi_{n+1}-2\psi_{n}+\psi_{n-1}\right)}^{2}\\ =\eta \left(\psi_{n+2}-3\psi_{n+1}+3\psi_{n}-\psi_{n-1}\right) \end{array}$$

where *ε* ≠ 0 is an auxiliary parameter [3]. The slopes at opposite ends of the solution characterizing a jump in the magnetic field across a current sheet differ, so force balance fails. It is when *η* is of the same order of magnitude as the mesh size that it becomes necessary to switch to the conservative method.

## 4. Bifurcated Equilibria in Tokamaks

The variational method employs a representation

B=∇s×∇θ=∇ϕ+ζ∇s

of the magnetic field in terms of the toroidal flux *s*, another flux function *θ*, and two Clebsch potentials *ϕ* and *ζ*. If there is no net current and *ι* stands for the rotational transform, the variables *θ* + *ιϕ* and *ϕ* can be renormalized to become invariant poloidal and toroidal angles on each flux surface *s* = const., where *s* is the radial coordinate. We refer to the Fourier coefficients *B_mn_* in the resulting expansion

$$\frac{1}{B^{2}}=\sum B_{mn}\left(s\right)\cos\left[m\theta -\left(n-\iota m\right)\phi \right]$$

as the magnetic field spectrum. There is an effective correlation between the Fourier coefficients Δ*_mn_* of the plasma boundary and the corresponding coefficients *B_mn_*. Good confinement is achieved for a QAS magnetic spectrum so the first column *B_m_*_0_ dominates, or has quasihelical symmetry (QHS) so the diagonal terms *B_mm_* dominate [15].

Solving the MHD equations for **J** and calculating its divergence yields an expansion

$$\frac{J\cdot B}{B^{2}}=p^{\prime }\sum \frac{mB_{mn}\left(s\right)}{n-\iota m}\cos\left[m\theta -\left(n-\iota m\right)\phi \right]$$

for the parallel current in stellarators like the one for the magnetic field strength. In three dimensions the small denominators *n* − *ιm* on the right are seen to vanish at a dense set of rational surfaces where *ι* = *n*/*m*. The presence of these resonances shows that smooth solutions of the equilibrium problem with 3D asymmetry do not in general exist. Hence one should only try to construct weak solutions that may not be differentiable [16]. Small magnetic islands become modeled computationally by contact discontinuities when conservation form is applied.

The NSTAB code has been developed to model 3D equilibrium and stability in tokamaks with a poloidal field provided by net plasma current or in stellarators with external coils for the poloidal field. A good simulation of the physics is obtained by using *s* as a radial coordinate and *θ* as an unknown and introducing a nested surface hypothesis to make *p* a prescribed function of *s*. Islands in bifurcated solutions of the tokamak problem suggest that 3D effects may be more important than has generally been realized. Remarkable examples of bifurcated equilibria are three-dimensionally asymmetric solutions of the tokamak problem subject to axially symmetric boundary conditions for which a 2D solution exists, too.

Fully 3D equilibria are calculated by first imposing and later releasing a suitable constraint in runs of the NSTAB code chosen to find bifurcated solutions that cannot be obtained without permitting discontinuous alterations in the topology of the magnetic surfaces [6]. On relatively crude radial grids the computations capture small islands whose widths are of the same order of magnitude as the mesh size, but are big enough to account for a significant change in topology. The residuals increase while a sensitive mode is being computed and only later decrease to a level of round off error establishing that the discrete problem has been solved numerically. Islands are found readily at a resonance midway between two mesh points because the finite difference scheme captures discontinuities there more effectively. The outcome of such computations is consistent with observations of NTMs and ELMs in laboratory plasmas.

We have computed a variety of bifurcated equilibria in tokamaks. The Kolmogoroff-Arnold-Moser theory displays small denominators at rational surfaces of 3D solutions, and analysis of the continuous spectrum shows that the stability of tokamaks is marginal [9]. These results are consistent with observations of sawtooth oscillations, NTM and ELM instabilities, and disruptions. Acceptable 3D solutions of the MHD equilibrium equations may not exist, may not be unique, and may not depend continuously on the data. Yet success of experiments fosters a belief that it is possible to design a magnetic fusion reactor. A QAS stellarator is a good alternate configuration to study, since some rotational transform should come from the external magnetic field. A productive approach to these issues lies in the computation of weak solutions of the magnetostatic equations.

## 5. Confinement and Quasineutrality

The Monte Carlo method evaluates transport by tracking test particles in a fixed background and calculating how long it takes them to leave the plasma. Agreement with experiment is achieved by computing the ion and the electron confinement times separately and introducing 3D effects to impose quasineutrality so that the results become the same. In simulations we compare observations of the energy confinement time with a third of the test particle confinement time. This theory becomes more convincing in light of the new 3D equilibria that can now be computed.

Our transport calculations employ the TRAN Monte Carlo code after applying the NSTAB code to find bifurcated equilibria that determine the magnetic field in a plasma background. The random walk approximating a collision operator need not conserve momentum in the split time calculation of the test particle model. Experimental data are used to validate the results of numerical computations. At temperatures of 3 keV observed in the Large Helical Device (LHD) experiment, the TRAN code predicts an energy confinement time of 160 ms, which agrees with measured values [11].

Runs of the TRAN code have been compared with measurements from tokamak experiments as well. These simulations suggest that a 2D model does not explain observations. The Monte Carlo method takes into account a long mean free path and the complex geometry of the drift surfaces, so it captures the essential physics. In the calculations, if 3D perturbations of 1/*B*^2^ are set equal to zero and no iteration of the electric potential is performed, then the confinement times do not come into line with observations. But when quasineutrality is successfully imposed to determine the 3D terms then the numerical answers agree with measured data [12]. Among many bifurcated equilibria that exist for tokamaks, only those satisfying a condition of quasineutrality seem to be valid. That can also be achieved by adjusting the collision frequency.

The numerical evidence shows that it is not realistic to assume intrinsic ambipolarity in tokamaks. The justification for that hypothesis must be examined from the point of view of more rigorous mathematical analysis. A simplified theory that neglects the role of the displacement current because it is multiplied by a small factor like the Debye length seems to ignore important contributions that appear when long orbits are tracked accurately by the Monte Carlo method. The computational model suggests that for the 2D tokamak problem there might be no steady state solution satisfying the requirement of quasineutrality.

The NSTAB code provides accurate information about the magnetic spectrum, together with current and pressure profiles. That enables the TRAN code to estimate the confinement times of both ions and electrons in a realistic fashion. The result of such calculations suggests that a failure of quasineutrality may lead to resonant perturbations in force balance that could trigger the appearance of bifurcated equilibria with 3D asymmetries affecting stability and transport. We examine these issues in a renewed attempt to account for anomalies in theoretical estimates of confinement time that are incompatible with observations. With special attention to the mathematics, we perform computational analyses of confinement in plasmas that treat transport as a random walk of test particles among complicated orbits and drift surfaces that have global structure.

## 6. Stability of Stellarators

One way to assess MHD stability of the toroidal equilibrium of a plasma by the variational method is to introduce a small perturbation in the equilibrium equations and then calculate the corresponding change of the solution. A reliable test of linear stability by means of the NSTAB code consists in removing the forcing term after a certain number of cycles, but continuing the iterations to find out whether a bifurcated solution different from the original one can be constructed. Failure of uniqueness usually furnishes a convincing proof of instability. The preconditioned method of steepest descent implemented in NSTAB produces a perturbation of the solution that incorporates more structure of the unstable mode than was input by the displacement. In this sense the code solves for the most dangerous eigenfunction of linear stability theory without requiring calculation of derivatives that may not exist. The good resolution of the spectral method, combined with excellent accuracy in the radial difference scheme, is what makes the approach a success in practice.

Runs of the NSTAB code show that the LHD stellarator is linearly unstable, but remains nonlinearly stable, at a pressure ratio *β* above 4.0 % achieved experimentally. Predictions of ballooning stability for the LHD are more pessimistic than these estimates from bifurcated solutions calculated over 1, 2, 5 or 10 field periods. In the case of tokamaks we have made detailed comparisons with observations from the DIII-D, and there is substantial agreement. Examination of 3D bifurcated equilibria that we have calculated confirms the experimental result that in a standard configuration the *β* limit is near 5 %, but when shaping coils are employed to push the plasma inward and reduce the aspect ratio then values of *β* above 10 % can be attained [13].

## 7. Power Plant Studies

We have described applications of the NSTAB and TRAN codes to tokamak and stellarator equilibria with parameters of major experiments. Good correlation of the computations with observations in the DIII-D and the LHD validates predictions about equilibrium, stability, and transport for QAS stellarators. A reactor configuration with two field periods has been designed that has major radius 8 m and plasma radius 3 m. The 3D asymmetries are below 0.005 in units of the field strength, the *β* limit is 4 %, and 12 moderately twisted coils provide robust magnetic surfaces with islands whose widths are small. The gap between the separatrix and filaments defining the coils is everywhere bigger than 140 cm, and there is access for maintenance through ports between the coils [14]. Theory and practice show that it is only when smooth coils are found that the structure of the field lines outside the plasma becomes well enough organized to allow for the construction of an effective divertor. That is achieved by employing a judiciously filtered set of harmonics in an implementation of the Biot-Savart law.

Refinement of the numerical method implemented in the NSTAB code produces a diagnostic for the presence of magnetic islands in tokamaks and stellarators. When this technique is applied to ITER, it suggests that bifurcated equilibria contribute to disruptions and degrade confinement. If the difficulties turn out to be significant one might want to switch to a similar QAS stellarator as the best concept to demonstrate properties of a fusion reactor [5]. Therefore NSTAB computations are needed to see whether the effect of islands is tolerable provided the rotational transform avoids dangerous resonances.

**Fig. 2:**
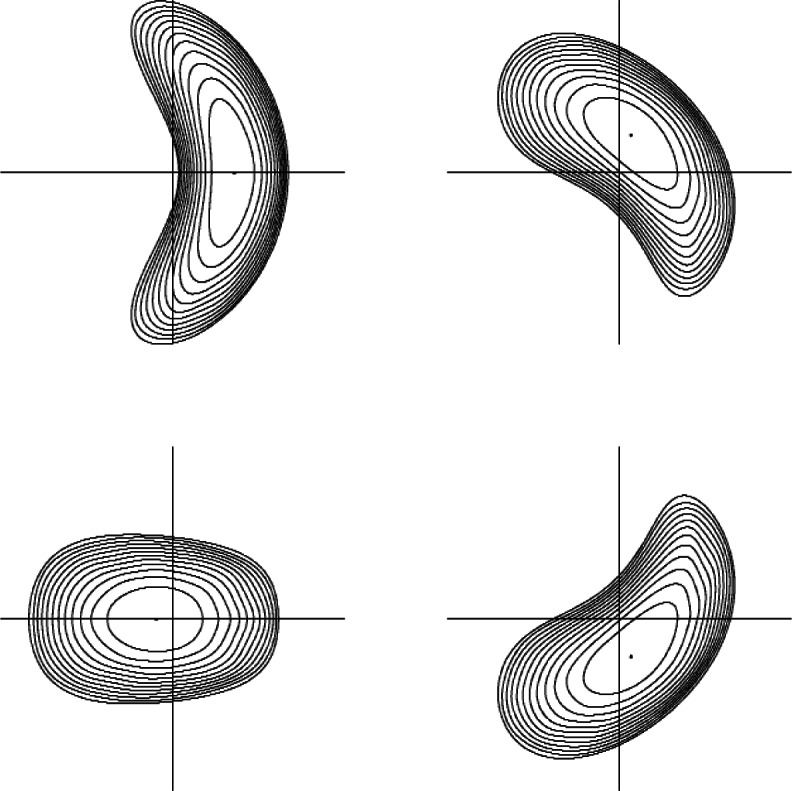
Flux surfaces at four cross sections over the two field periods of a stellarator equilibrium at *β* = 0.03. For toroidal flux in the interval 0 < *s* < 1 we have pressure *p* = 0.029 (1 – *s*^2^) ^1.5^ and rotational transform *ι* = 0.47 – 0.49(0.6 − *s*) ^2^. This configuration is a good candidate for DEMO because its geometry is similar to that of the ITER tokamak. There is enough flexibility in the shaping of the plasma to control the stellarator contribution to *ι* so as to compensate for unforeseen complications with the bootstrap current.

For realistic assumptions about the bootstrap current and with a sensible choice of modular coils, we look for 3D configurations that perform well at reactor conditions [7]. Figure 2 displays a candidate with geometry like that of ITER. This numerical solution of the MHD equations has converged to the level of round off error in double precision, assuring existence, uniqueness, and stability. It remains to find a way to build the coil structure that is not inordinately expensive. One possibility is a sturdy vacuum vessel with grooves to support the coils.

Progress in magnetic fusion research calls for a major experiment to show that the ion temperature in a stellarator can be raised to the level necessary for fusion. Because questions remain open about the feasibility of a fusion reactor, it is important to keep in mind the advantages available from fully 3D geometry, even if that adds to the cost of electricity. The two-dimensional model of tokamaks used to defend ITER is inadequate. Better 3D simulations produced by the NSTAB and TRAN computer codes predict that the project is at risk. A compact stellarator configuration could be employed in DEMO to correct the problem, but additional information would be needed from experiments with the QAS and QHS concepts. Wide variations in the length and time scales occurring in the plasma physics of magnetic fusion make it hard to describe the dynamics of stellarators and tokamaks by solving conventional differential equations numerically, so our success in predicting the performance of major fusion experiments by running the NSTAB and TRAN computer codes merits more attention.

## 8. The QAS Stellarator Project

Implementation of the quasisymmetric stellarator idea requires choosing the shape factors Δ*_mn_* of the plasma boundary so that the matrix *B_mn_* of spectral coefficients becomes dominated by a single column for QAS configurations or the principal diagonal for QHS configurations. Success depends on the fortunate circumstance that when the quotient of the aspect ratio divided by the number of field periods is near 2, then the value of the Fourier coefficient *B*_21_ producing the bulk of the rotational transform becomes remarkably small. The best candidates for a reactor seem to be either QAS stellarators with two field periods or QHS stellarators with five periods. Failure of a QAS experiment at the Princeton Plasma Physics Laboratory was partially due to a choice of three field periods and low aspect ratio producing inadequate quasisymmetry, combined with belief in local stability criteria that are too pessimistic and lead to unnecessarily complicated geometry.

Recent observations of surprisingly high values of *β* in the LHD experiment have shown that predictions of the *β* limit in stellarators by the Mercier and ballooning mode criteria are too low [11]. More realistic results are obtained by running the NSTAB equilibrium and stability code to look for multiple solutions of the MHD equations that are typical of instability. These computer simulations have been validated by extensive comparisons with observations. The numerical method is facilitated by computational evidence establishing that with our choice of indices, and with the exception of some resonant cases, the Fourier coefficient *B_mn_* in the spectrum is largely determined by the corresponding coefficient Δ*_mn_* in the formula for the boundary of the plasma. Experience with this procedure establishes that as few as 20 parameters Δ*_mn_* may suffice to specify an optimal configuration. After the shape of the plasma has been determined, Fourier series filtered to match those for the separatrix can be selected judiciously to define an external magnetic field with robust flux surfaces adequate for an experiment.

Specifications are given in Fig. 2 and Table 1 for a QAS stellarator with two field periods of the kind we have described. The results summarize a study that would not be easy to reproduce without this data. The configuration itself is important for the role it might play at some future date in the design of DEMO, which should have at least some rotational transform coming from an external magnetic field so that *ι* > 0 at the edge of the plasma. The high cost of the LHD experiment in Japan shows that a proof of principle experiment to see whether a compact QAS stellarator like this could achieve the values of *β* or of the hydrogen ion temperature required for a fusion reactor would be very expensive.

A less ambitious experiment could be constructed much more easily. With a major radius of just 2 m and a magnetic field of only 0.5 tesla, one should be able to address engineering issues about construction of the coils and one should be able to find out whether the bootstrap current necessary for good performance of a compact stellarator will materialize. For an application to DEMO the QAS stellarator has the advantage that the geometry and in particular the aspect ratio are close to those of ITER.

We have described how the TRAN Monte Carlo code estimates energy confinement time by performing independent test particle computations of ion and electron confinement times and imposing quasineutrality by choosing the radial electric field to make them agree. The dependence of the guiding center orbit equations on the magnetic spectrum explains why QAS stellarators perform well as candidates for construction of a fusion reactor. In 2D trapped particles are well confined, whereas in fully 3D geometry they are subject to prompt losses. The configuration specified in Table 1 has a very accurate quasisymmetry with 3D contributions below half a percent of the magnetic field strength. The exceptionally low level of asymmetry in a device with the aspect ratio *A* = 2.5 makes it attractive as a power plant. These concepts apply also to the ITER tokamak.

The numerical calculations suggest that QAS stellarators have good thermal confinement for a fusion power plant, but significant difficulties remain about the prompt loss of hot alpha particles in both tokamaks and stellarators that raise serious questions in materials science. However, the excellent quasisymmetry of the configuration specified in Table 1 does produce a remarkable reduction in these losses.

## Figures and Tables

**Table 1:** Fourier coefficients Δ*_mn_* defining the QAS stellarator in Fig. 2 for values *m* = −1, 0, 1, 2, 3, 4 of the poloidal index and values *n* = − 1, 0, 1, 2, 3, 4 of the toroidal index. This is a configuration that depends on bootstrap current for good performance

*m*\*n*	−1	0	1	2	3	4
−1	0.200	0.140	0.000	0.000	0.000	0.000
0	0.000	1.000	0.006	0.007	0.000	0.000
1	0.042	2.500	0.084	0.006	−0.002	0.000
2	0.010	−0.100	−0.350	−0.058	−0.010	0.000
3	0.000	0.000	0.030	0.050	0.017	0.004
4	0.000	0.000	0.025	−0.020	0.000	0.000
